# Draft Genome Sequence of Sinorhizobium meliloti Strain CXM1-105

**DOI:** 10.1128/MRA.01621-18

**Published:** 2019-01-10

**Authors:** Olga A. Baturina, Victoria S. Muntyan, Alexey M. Afonin, Maria E. Cherkasova, Boris V. Simarov, Marsel R. Kabilov, Marina L. Roumiantseva

**Affiliations:** a Institute of Chemical Biology and Fundamental Medicine, Siberian Branch of the Russian Academy of Sciences (ICBFM SB RAS), Novosibirsk, Russia; b All-Russian Research Institute for Agricultural Microbiology (ARRIAM), Laboratory of Genetics and Selection of Microorganisms, Saint Petersburg, Russia; Louisiana State University

## Abstract

Sinorhizobium meliloti is a Gram-negative bacterium which fixes atmospheric nitrogen in symbiosis with Medicago spp. We report the draft genome sequence of S. meliloti strain CXM1-105, associated with nodules of Medicago sativa subsp. varia (Martyn) Arcang.

## Abstract (precis)

Sinorhizobium meliloti is a Gram-negative bacterium which fixes atmospheric nitrogen in symbiosis with Medicago spp. We report the draft genome sequence of S. meliloti strain CXM1-105, associated with nodules of Medicago sativa subsp.

## ANNOUNCEMENT

Sinorhizobium meliloti CXM1-105 is a UV-light-obtained mutant of strain CXM1, which is a spontaneous streptomycin-resistant mutant of commercial strain 425a, which was recovered from Medicago sativa subsp. varia nodules in Kazakhstan (1 – 3). CXM1-105 has been used as a reference strain in plant tests and as a model strain in genetic experiments (1 – 4).

A single colony of CXM1-105 was grown overnight in tryptone-yeast extract (TY) broth (28°C, 180 rpm shaking) (5). Genomic DNA (gDNA) was isolated using a phenol-chloroform extraction method (6). Part of the gDNA was sheared in a microTUBE AFA fiber snap-cap tube using a Covaris S2 instrument in order to obtain fragments of about 600 bp. The paired-end library was prepared using a NEBNext Ultra II DNA library prep kit for Illumina (NEB) and dual-index NEBNext multiplex oligos (NEB). Whole-genome sequencing of the CXM1-105 library was conducted with reagent kit version 3 (2 × 300 bp) on a MiSeq genome sequencer (Illumina) at the Genomics Core Facility (ICBFM SB RAS). A total of 1,142,000 reads were generated. Adapter and low-quality sequences were removed using bbduk (ktrim=r k = 23 mink = 11 hdist = 1 tpe tbo minlen = 25 qtrim=rl trimq = 10) (7). Long reads were generated using a MinION sequencer (Oxford Nanopore) in ARRIAM. gDNA was used for the construction of a barcoded DNA library according to the 1D native barcoding genomic DNA (with EXP-NBD103 and SQK-LSK108) protocol. Albacore version 2.3.1 was used to base call the raw fast5 files. The run yielded 337,584 reads comprising 1.9 Gbp. The reads were demultiplexed using Deepbinner (8) and further cleaned using Porechop (https://github.com/rrwick/Porechop), both with default parameters. A total of 11,067 reads with an *N*_50_ value of 12,114 bp comprising 77 Mbp were attributed to the strain CXM1-105.

Illumina and Nanopore reads were assembled using Unicycler version 0.4.6 (9) with conservative mode, yielding 10 contigs. The coverage was 42× for Illumina and 11× for Nanopore reads. Contig alignment against the Rm1021 genome using progressiveMauve (version 20150226) (10), with default settings, resulted in 8 of 10 contigs belonging to the chromosome (3,635,790 bp; 62.8% GC content; GenBank accession number AL591688) and two others corresponding to plasmids SMa (843,540 bp; 62.6% GC content; GenBank accession number AE006469) and SMb (1,659,814 bp; 62.4% GC content; GenBank accession number AL591985). The NCBI Prokaryotic Genome Annotation Pipeline (11) was used for genome annotation, and 6,706 protein-coding genes, 3 rRNA operons, 54 tRNAs, and 1 transfer-messenger RNA (tmRNA) were identified in the CXM1-105 genome. It should be noted that Islander algorithm (12) revealed only one genomic island (GI) which was not similar to the three GIs of Rm1021 (GenBank accession number AL591688) (13). The GI (10.9 kbp, 58.0% GC content, and 9 open reading frames [ORFs]) associates with tRNA-Lys (DA101_001295) and contains 3 ORFs (DA101_001315, DA101_001320, and DA101_001340) which are HK97 phage family proteins.

### Data availability.

The genome sequence of Sinorhizobium meliloti CXM1-105 has been deposited in GenBank under the accession number PZMJ00000000. Raw sequencing data sets have been registered in the NCBI SRA database under accession number SRS3875810. This announcement describes the second version of the genome assembly.
